# Evaluation of 10 genotypes of sweetpotato for fries

**DOI:** 10.1002/fsn3.881

**Published:** 2019-01-23

**Authors:** Damian Laryea, Debora Koomson, Ibok Oduro, Edward Carey

**Affiliations:** ^1^ Department of Food Science and Technology KNUST Kumasi Ghana; ^2^ International Potato Center (CIP) Fumesua Ghana

**Keywords:** beta‐carotene, browning index, frying, sweetpotato fries, vitamin A

## Abstract

The potential use of selected genotypes of sweetpotato for fries was evaluated. Moisture, fat, browning index, and beta‐carotene content of fries from 10 sweetpotato genotypes, *Apomuden, Bohye, Nanungungu, Otoo, CIP442162, Patron, CIP440390, Obare, Dadanyuie*, and *Tu‐Purple*, were determined by standard methods. Sensorial properties of the fries were further evaluated by an in‐house trained panel of eight members using standard methods. Fat content was highest in *CIP442162* and low in *Dadanyuie*, while beta‐carotene content decreased by 44.27% in *Bohye* and 13.20% in *Nanungungu* after frying. Browning index was highest in the orange‐fleshed and purple genotypes, but this was mostly due to their flesh colors and not the frying conditions. Fries from orange‐fleshed genotypes, *Apomuden* and *Nanungungu,* were considered to be sweeter than the other genotypes. Detection of caramel and starch (rawness sensation) was very low for all genotypes assessed. Oily mouthcoat, moistness, and sogginess were detected in mostly the orange‐fleshed genotypes, particularly *Apomuden* and *Nanungungu*. *Tu‐Purple*,* Bohye*, and *CIP440390* produced moderately crunchy fries and had the highest score for desirable attributes compared with the other genotypes. *Tu‐Purple*,* Bohye*, and *CIP440390* could be explored in commercial production of fries for enhanced utilization of developed sweetpotato genotypes.

## 1 INTRODUCTION

Sweetpotato (*Ipomoea batatas* L.) is a dicotyledonous plant, belonging to the morning glory family Convolvulaceae (Watson & Dallwitz, 2000). They are large root vegetables that are starchy and generally have a sweet taste. Sweetpotato is the seventh‐ranked food crop in the world following wheat, rice, maize, potato, barley and cassava (Tan, 2015). It is a productive and profitable crop and is able to adapt successfully to a wide range of habitats including marginal environments (Aina, Falade, Akingbala, & Titus, 2009). Compared to most other root and tuber crops, sweetpotato also has positive properties such as short production cycle, increased nutritional value, and sensory versatility in terms of flesh color, texture, and taste (Truong & Avula, 2010). Artificial selection and the occurrence of natural hybrids and mutations of sweetpotato have led to the existence of a large number of cultivars with a range of physical and chemical properties which affect appearance and functional properties (Aina et al., 2009).

As an important economic crop, over 104 million tons of sweetpotatoes is produced across the world (FAOSTAT, 2008). Central America and the western coast of South America are the original home of sweetpotato (Tan, 2015). However, sweetpotato is largely cultivated in China with more than 3.5 million hectares which makes up 43% of the world total (Tan, 2015). Ghana produced 131,990 metric tonnes of sweetpotato in 2012 according to MoFA (2013).

In Ghana, sweetpotatoes are mostly fried or boiled when yams are either out of season or are dried (Danquah, Doku, & Boakye, 2000). They can be processed into different food products which include confectionery, snacks, noodles, breads, chips, and fries (Kimberly, 2015). Sweetpotato genotypes with increased amounts of provitamin A (beta‐carotene) and ascorbic acid will have an added advantage of nutrition when used in snack foods. In the United States, there has been an increase in consumer demand for sweetpotato fries and many processing companies are venturing into its production (U.S. Sweetpotato Council, 2013). This has led to the development of new genotypes that possess qualities suitable for the production of fries as part of the sweetpotato breeding program of the US Sweetpotato Council.

In Ghana, several genotypes of sweetpotato including orange‐fleshed sweetpotatoes (high in beta‐carotene) have been selected by the CSIR‐Crops Research Institute breeding program; however, their potential use as fries is yet to be investigated. It is necessary to understand the characteristics of the sweetpotatoes produced and their potential use for fries, in order to inform processors and consumers on which genotypes to process into fries.

Dry matter content in sweetpotato which mainly consists of carbohydrate is very important for the selection of a particular genotype for fries and contributes to the taste and texture of the product (Gao et al., 2014). As with potato, it may be important to look out for superior characteristics in dry matter, good product color, lower oil absorption, and increased crop yield (Abong, Okoth, Imungi, & Kabira, 2010) to produce fries preferred by consumers, and that will also increase sales for sweetpotato farmers.

In spite of its great potential, the consumption of the sweetpotato root is still limited in Ghana although it is regularly being promoted (Adu‐Kwarteng, Otoo, Osei, & Baning, 2002). In Ghana, the crop is given less attention in our menu planning as compared to other roots and tuber such as yam, cassava, or cocoyam reportedly because of its sweetness. Low consumption of sweetpotato, which both reflects and results in low demand and low commercial value, can be attributed to the poor utilization of sweetpotato in popular food products that have mass‐market appeal, and its lack of use in large industrial applications. This study therefore seeks to evaluate 10 genotypes of sweetpotato to select the most suitable to be used as fries, which present a growing market both in Ghana and globally.

## 2 MATERIALS AND METHODS

### 2.1 Source of raw materials and determination of starch and dry matter

The 10 different genotypes (*Bohye, Tu‐Purple, Nanungungu, Dadanyuie, Apomuden, CIP440390, CIP442162, Patron, Otoo,* and *Obare*) of sweetpotato were obtained from the fields of the sweetpotato breeding program of the International Potato Center (CIP) and CSIR‐Crops Research Institute, Ghana. Yam, which was also used as a control, was obtained from Ayigya market center, Kumasi, Ghana. Dry matter and starch content of samples were determined with reference to methods by Kathabwalika, Chilembwe, and Mwale (2016).

### 2.2 Frying of the roots

Four sweetpotato storage roots for every genotype were selected at random, washed under running potable tap water, and manually peeled using a stainless sweetpotato peeler. The roots were then cut manually into a French‐fry strip (0.7 cm × 0.7 cm × 7.5 cm) using a stainless steel cutter. The cut strips were rinsed under running tap water and patted with tissue paper to remove surface water and deep‐fried in sunflower oil (2.5 L) at 175°C for 5 min using a domestic fryer (Akai Deep Fryer, model DF006A‐388; China). An average of 15 strips were fried for each batch of sweetpotato genotype. Fries obtained were placed in ziplock bags and then used in moisture, fat, color, and beta‐carotene analysis. Sensory evaluation was conducted within 30 min after frying.

### 2.3 Sensory evaluation of fried samples

Quantitative descriptive analysis was conducted using eight trained panelists. Panel members were trained for three times within a week for 2 weeks. They were taken through basic tasting for sweetness, sourness, and bitterness using sugar solution, lime juice, and quinine at different concentrations, respectively. Afterward, they were taken through texture and color training using fruits, crackers, and crisps. Fried sweetpotatoes were introduced to participants, and they were asked to come up with attributes that can best describe the fried sweetpotatoes or fries in general. A scale was designed based on these attributes, and panelists were asked to assess the fried sweetpotatoes. The attributes were color, crunchiness, hardness, moisture, sogginess, caramel, starch/rawness, and oily mouthcoat, all on a scale of 1–9 with 9 indicating the highest and 1 the lowest. The evaluations were done between the hours of 10 a.m. and 12.30 p.m. at the International Potato Center (CIP‐Ghana) postharvest laboratory at CSIR‐Crops Research Institute.

### 2.4 Determination of moisture, fat, color, and beta‐carotene of sweetpotato fries

#### 2.4.1 Determination of moisture and fat content

Moisture and fat content were determined as described by AOAC (1990).

#### 2.4.2 Determining the color of sweetpotato fries

A handheld Konica chromameter CR‐410 (Minolta Co. Ltd., Osaka, Japan) was used to determine the color of milled sweetpotato fried samples (Oner & Wall, 2013).

#### 2.4.3 Beta‐carotene analysis on fresh and fried sweetpotatoes

The β‐carotene content of the orange‐fleshed sweetpotato genotypes (*Apomuden, Bohye*, and *Nanungungu*) was determined using the method described by Imungi and Wabule (1990). The Shimadzu HPLC equipment used was made up of an LG6 pump, a UV–Visible detector, a CR6 recorder, ODS Reversed‐Phase column, and a Rheodyne 1725 injector. Mobile phase was made up of 70% acetonitrile, 20% dichloromethane, and 10% methanol. The flow rate was also 1 ml/min and wavelength 450 nm.

### 2.5 Statistical analysis

Data obtained were analyzed using one‐way analysis of variance (ANOVA) and Tukey's HSD test at 95% confidence interval. Data were expressed as mean and standard deviation. Pearson's correlation was also employed to ascertain any relationship between parameters determined.

## 3 RESULTS AND DISCUSSION

### 3.1 Dry matter and starch of sweetpotato roots

#### 3.1.1 Dry matter of sweetpotato roots

Dry matter refers to materials remaining after removal of water. The dry matter of the different genotypes of sweetpotato ranged from 24.05% to 44.99%. *Apomuden,* an orange‐fleshed sweetpotato genotype, had the lowest dry matter content (24.05%), while *Dadanyuie* had the highest value (44.99%) (Table 1). These were similar to the OFSP values (23.3%–34.45%) reported by Kathabwalika et al. (2016), though our value for *Dadanyuie* was at the upper limits reported for sweetpotato. The cultivar (BV/009) that recorded the lowest value according to Kathabwalika et al. (2016) was an orange‐fleshed cultivar. Dry matter is a very essential quality attribute in the production of sweetpotatoes as it shows mealiness in boiled or the core of fried sweetpotato and it is an attribute that is mostly preferred by consumers (Kathabwalika, Chilembwe, Mwale, Kambewa, & Njoloma, 2013).

**Table 1:** Dry matter and starch content of sweetpotato genotypes

Genotype	Starch content (%)	Dry matter (%)
*Bohye*	19.79 ± 0.06^f^	32.97 ± 0.26^abc^
*CIP442162*	17.59 ± 0.13^e^	29.54 ± 0.58^ab^
*Patron*	17.46 ± 0.20^e^	36.31 ± 0.24^abc^
*Obare*	16.50 ± 0.08^d^	31.03 ± 0.17^ab^
*Nanungungu*	15.87 ± 0.06^c^	29.97 ± 0.26^ab^
*Tu‐Purple*	14.46 ± 0.01^b^	33.77 ± 0.25^bc^
*Otoo*	14.18 ± 0.15^b^	36.08 ± 0.09^bc^
*Dadanyuie*	14.11 ± 0.04^b^	44.99 ± 0.75^c^
*CIP440390*	14.09 ± 0.05^b^	32.13 ± 0.20^abc^
*Apomuden*	10.12 ± 0.03^a^	24.05 ± 0.36^a^

*Notes*

Values are represented as mean ± standard deviation.

Values in the same column with the same superscripts are not significantly different (*p* > 0.05).

The results presented in this study fell within the general dry matter content of sweetpotato cultivars, which ranges from 13% to 45% (Aina et al., 2009; Mensah, Ibok, Ellis, & Carey, 2016). The dry matter content (24.05%) of the orange‐fleshed genotype (*Apomuden)* also fell between 20.4% and 27.8% as presented by Hagenimana, Carey, Gichuki, Oyunga, and Imungi (1998) of other orange‐fleshed sweetpotato genotypes. However, the value of the purple‐fleshed (*Tu‐Purple*) genotype did not fall within the range (20.3% to 30.2%) reported by Hagenimana et al. (1998), but was slightly higher (33.77%).

Waramboi, Dennien, Gidley, and Sopade (2011) had dry matter content values ranging from 14.7% to 28.2%. These values are however lower than the results of this study. The orange‐fleshed genotypes recorded the lowest dry matter content values ranging from 18.2% to 22.3% (Waramboi et al., 2011). This is however a little lower than the values recorded for our orange‐fleshed genotypes (Table 1). No significant differences (*p* > 0.05) were observed between the orange‐fleshed genotypes, *Apomuden* and *Nanungungu*. Moreover, there were no significant differences (*p* > 0.05) observed among some pale yellow, cream genotypes and the orange‐fleshed genotypes (Table 1).

#### 3.1.2 Starch content of sweetpotato roots

In relation to the dry matter content, the starch content followed a similar trend. *Apomuden*, an orange‐fleshed genotype, had the lowest starch content of 10.12%, while *Bohye* had the highest (19.79%), on fresh weight basis (Table 1). Although *Nanungungu* is an orange‐fleshed genotype, it had a much higher starch content than *Apomuden*. This may be due to their genetic makeup. *Nanungungu* is a landrace genotype already grown in the Upper East Region of Ghana and has been known to be one of the few orange‐fleshed genotypes with relatively higher starch content and dry matter. Such types were reported in East Africa by Tumwegamire et al. (2011). *Apomuden*, on the other hand, was made to contain very high beta‐carotene content; however, it ended up with much higher moisture content and very low dry matter and starch. This may result in very soft fries. A significant difference was observed between *Apomuden* and all other genotypes in terms of the starch content; however, no significant differences were observed among Tu‐Purple, Otoo, *Dadanyuie*, and CIP440390. *Nanungungu* was also found to be significantly different from all other genotypes (Table 1).

The values obtained were lower than the values recorded by Kathabwalika et al. (2016). The starch content of the genotypes in their study was in the range of 22.4% to 27.7%. Their study also revealed that the average starch content of the genotypes across all the sites were in the range of 20.5% to 29.6%. Utomo and Rahman (2015) reported starch content to be significantly different (*p* < 0.05) among three cultivars (white, yellow, and orange). The starch content values ranged from 12.34% to 19.30%. These values fall within those obtained in this study. Utomo and Rahman (2015) reported that the white cultivar had the highest starch content (19.30%) which is however higher than the values obtained for the white‐fleshed genotypes in this study (Table 1). The pale yellow‐fleshed genotype was rather found to have the highest starch content value in this study. However, the values recorded by Tuffuor (2013) were found in the range of 14.6% to 31.7% (fwb), which was comparably higher than what was observed in this study. The combination of high dry matter (>25%) and starch helps in selection of genotypes for use as fries (Lebot, 2009).

### 3.2 Moisture and fat content of fries

#### 3.2.1 Moisture content of fries

The results revealed high moisture content in the fried sweetpotato genotypes. Moisture content ranged from 20.78% to 51.68%, with *Apomuden* having the highest moisture content value while *CIP440390* had the lowest moisture content among the 10 genotypes (Table 2). However, yam, which was used as a control, recorded the least moisture content with a 10.79% value. Genotypes that had low dry matter content seemed to have higher moisture content, and those with higher dry matter content also had low moisture content after frying. This is true because dry matter increases with decrease in moisture content and vice versa.

**Table 2:** Moisture and fat content of the sweetpotato fries

Genotypes	Moisture content (%)	Fat content (%)
*Yam*	10.79 ± 0.02^a^	20.38 ± 3.45^abc^
*CIP440390*	20.78 ± 0.20^b^	23.54 ± 1.39^bc^
*Obare*	21.35 ± 0.73^b^	14.25 ± 1.21^a^
*Patron*	22.24 ± 1.09^bc^	18.54 ± 0.01^abc^
*Tu‐Purple*	25.25 ± 0.21 ^cd^	16.93 ± 2.43^ab^
*CIP442162*	25.54 ± 2.04^d^	23.96 ± 2.56^c^
*Otoo*	27.24 ± 0.08^de^	14.95 ± 1.63^a^
*Bohye*	29.68 ± 0.65^e^	17.96 ± 0.93^abc^
*Nanungungu*	33.43 ± 0.42^f^	17.04 ± 1.48^abc^
*Dadanyuie*	34.06 ± 0.94^f^	14.00 ± 0.23^a^
*Apomuden*	51.68 ± 0.13 ^g^	17.39 ± 0.76^abc^

*Notes*

Values are represented as mean ± standard deviation.

Values in the same column with the same letters are not significantly different (*p* > 0.05).

Truong et al. (2014) reported that moisture content of Covington sweetpotato French fries was in a range of 50.1%–67.7% depending on pretreatments and frying time. This is similar with the orange‐fleshed sweetpotato (*Apomuden*) fries which recorded 51.68% moisture content. The moisture content of restructured sweetpotato sticks made from white‐ and yellow‐fleshed sweetpotato cultivars reported by Utomo and Rahman (2015) was higher than that of the orange‐fleshed sweetpotato. This however contradicts the results obtained from this study with orange‐fleshed sweetpotato (*Apomuden*) recording the highest value (51.68%). This could be due to the genetic makeup of their genotypes and restructuring of the sweetpotato sticks. *Apomuden* has very high moisture content; however, the orange‐fleshed genotype reported by Utomo and Rahman (2015) may have had much higher dry matter than their white‐ and yellow‐fleshed sweetpotato genotypes reported in their study.

*Bohye* and *Nanungungu*, which are also orange‐fleshed genotypes, had values lower than that of the orange‐fleshed cultivar reported by Utomo and Rahman (2015). Odenigbo, Rahimi, and Ngadi (2012) also reported moisture content values ranging from 23.50% to 52.67% for French fries from five different cultivars of sweetpotato. Crispiness and increased shelf stability of fried products are mostly due to low moisture content (Fetuga, Odulaja, & Adelekan, 2013). Therefore, it is expected that yam may be more crunchy/crispy and may have a longer shelf life than the sweetpotato genotypes. Among the sweetpotato genotypes, the orange‐fleshed genotypes (*Apomuden*,* Nanungungu*, and *Bohye*) may be relatively less crunchy and may have a shorter shelf life.

Between genotypes, some of the genotypes were not significantly different (*p* > 0.05) from each other (Table 2). Oner and Wall (2012) recorded higher values of moisture content in purple‐fleshed sweetpotato (PFSP) fries. The values ranged from 20.13% to 60.04%. The moisture content of the purple‐fleshed sweetpotato genotype of this study was found in this range.

#### 3.2.2 Fat content of fries

Fat content ranged from 14.00% to 23.96%, with *Dadanyuie* having the lowest value while *CIP442162* had the highest value (Table 2). These values are higher than the fat content values (6.90%–15.54%) reported by Odenigbo et al. (2012) in their study. However, fat content in this study was found to be lower than that recorded by Esan, Sobukola, Sanni, Bakare, and Munoz (2015). These differences could be as a result of the different sweetpotato genotypes used.

Statistically, a number of sweetpotato genotypes were not significantly different from one another (*p* > 0.05) (Table 3). Fetuga et al. (2013) recorded higher values of fat content in fried sweetpotato crisps after pretreating the samples and frying at 170°C for 3 min. Their values ranged from 18.5% to 32.0% and were found in the range of 22.74% to 35.63%, reported by Rani and Chauhan (1995), but lower than 35.77% to 39.44% (Kita, 2002), both for potato crisps. The higher values may be due to the slice size of sweetpotatoes and genotype. Fat uptake during deep fat frying of French fries is usually affected by pretreatment and frying time (Lamberg, Hallstrom, & Olsson, 1990). The values could not have been the same considering the conditions under which the genotypes were produced.

**Table 3:** Color of sweetpotato fries

Samples	Color parameters					
	L*	a*	b*	c*	H	BI
*Nanungungu*	62.94 (0.03)d	14.24 (0.07)a	37.01 (0.04)a	39.66 (0.06)a	68.95 (0.08)b	21.80 (0.07)h
*Obare*	71.52 (0.11)h	3.02 (0.03)b	17.63 (0.02)b	17.89 (0.02)b	80.28 (0.08)e	5.47 (0.04)c
*Dadanyuie*	68.84 (0.05)f	3.71 (0.01)c	19.25 (0.01)c	19.60 (0.01)c	79.10 (0.02)d	6.63 (0.01)e
*Apomuden*	59.18 (0.01)b	15.55 (0.03)d	33.65 (0.05)d	37.07 (0.06)d	65.20 (0.01)a	24.04 (0.04)i
*Bohye*	76.02 (0.07)i	0.03 (0.00)e	19.05 (0.04)e	19.05 (0.04)e	89.92 (0.02)g	2.50 (0.00)a
CIP442162	70.58 (0.01)g	1.19 (0.01)f	25.89 (0.01)f	25.92 (0.01)f	87.38 (0.01)f	4.87 (0.00)b
Tu‐Purple	43.97 (0.04)a	14.58 (0.02)g	−2.87 (0.00)g	14.85 (0.01)g	348.87 (0.04)h	21.85 (0.02)h
CIP440390	67.89 (0.01)e	3.46 (0.01)h	16.45 (0.01)h	16.81 (0.01)h	78.15 (0.02)c	6.05 (0.01)d
Otoo	62.77 (0.01)d	4.91 (0.01)i	28.33 (0.00)i	28.75 (0.00)i	80.17 (0.02)e	10.13 (0.02)f
Patron	62.58 (0.01)c	5.41 (0.01)j	28.13 (0.02)j	28.64 (0.02)i	79.12 (0.04)d	10.69 (0.01)g

*Notes*

BI: browning index.

Values are represented as mean (standard deviation).

Values in the same column with the same letters are not significantly different (*p* > 0.05).

It was suggested that the increased surface moisture content resulted in an increased fat uptake (Lamberg et al., 1990). In this study, the moisture on the surface of the strips of sweetpotato was patted dry, and this may have influenced the absorption of oil.

Oner and Wall (2012) also reported fat content between 9.5% and 37.9%, dry weight, in purple‐fleshed sweetpotato French fry. Oil content in white‐fleshed potato French fries normally ranges from 9% to 15% (Miranda & Aguilera, 2006; Van Loon et al., 2007). The fat content of the white‐fleshed sweetpotato in this study fell within this range (Table 2).

High moisture and fat content may result in fries becoming soggier, and thereby less crunchy. This may not be desired by consumers. However, if fat content is high but moisture content is low, as can be seen in the yam in this study, the sample becomes crunchy and is desirable.

### 3.3 Sensory evaluation of sweetpotato fries

#### 3.3.1 Taste score for sweetpotato fries

**Figure 1:**
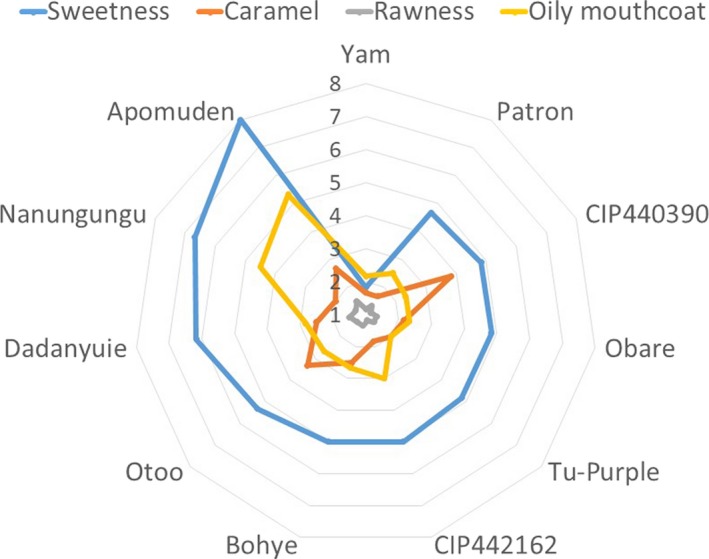
Taste score for sweetpotato fries

The taste score for the sweetpotato fries is made up of attributes such as sweetness, caramel, starch/rawness, and oily mouthcoat. These were grouped under taste score because they are all attributes perceived by means of taste. Of all the genotypes used in this study, the orange‐fleshed genotypes *Apomuden* and *Nanungungu* were the sweetest; with scores ranging from 7 to 8 (i.e., from sweet to very sweet) (Figure 1). The least sweet was the control sample, yam, while the less sweet among the sweetpotato genotypes were *Patron*,* CIP440390, Obare*,* Tu‐Purple*,* CIP442162*, and *Bohye*. These genotypes all had similar scores within 4 and 5, which indicates moderate sweetness (Figure 1).

Caramel sensation indicated the level at which the strips were burnt due to the frying process. A high detection of caramel sensation on the scale from 7 to 9 indicates that the sample got burnt, while a lower value of 1–6 indicates the sample was moderately burnt or did not burn at all. All samples had low scores with a slight detection of caramel sensation in *CIP440390*,* Bohye*,* Dadanyuie*, Otoo, and *Apomuden* (Figure 1). This indicates the frying conditions chosen were suitable for the strips. This could also be confirmed by the almost zero detection of starch/rawness in the samples (Figure 1). This indicated that the starches in the samples were gelatinized. Therefore with the slight detection of caramel sensation and almost no detection of starch/rawness, the frying conditions chosen were right for these strips. The frying conditions were chosen from a series of experiments with varying oil temperatures and time of frying.

Oily mouthcoat sensation was detected more in *Apomuden* and *Nanungungu* than in all the other genotypes. They had scores between 4 and 5, which indicates either slight detection of oily mouthcoat or no detection (Figure 1). These genotypes are the orange‐fleshed genotypes among the sweetpotato samples chosen for this study. According to Costa, Oliveira, and Boutcheva (2001), high oily mouthcoat may be due to the migration of oil into the cells of the food formed by cell wall shrinkage and water evaporation. Moreover, orange‐fleshed sweetpotato genotypes absorbing lots of oil during deep frying may be mainly due to the low starch or dry matter content and beta‐carotene content in the samples. Beta‐carotene is more nonpolar and easily dissolves in nonpolar solvents such as oil. Since the orange‐fleshed genotypes have more beta‐carotene as part of their components, interconnected with other components in the sample, they are more likely to absorb more oil than the non‐orange‐fleshed genotypes. *Bohye* is a pale orange‐fleshed genotype but has more starch or dry matter than *Nanungungu* and *Apomuden*, and hence may not have absorbed that much oil. However, these claims are not directly in sync with the fat content of the samples in Figure 1. For instance, yam, *CIP440390*, and *CIP442162* had the highest fat content; however, they had low oily mouthcoat values when sensory evaluation was conducted. This could be because oily mouthcoat may be enhanced by certain factors such as moisture content of sample or low starch content.

#### 3.3.2 Appearance score of sweetpotato fries

**Figure 2:**
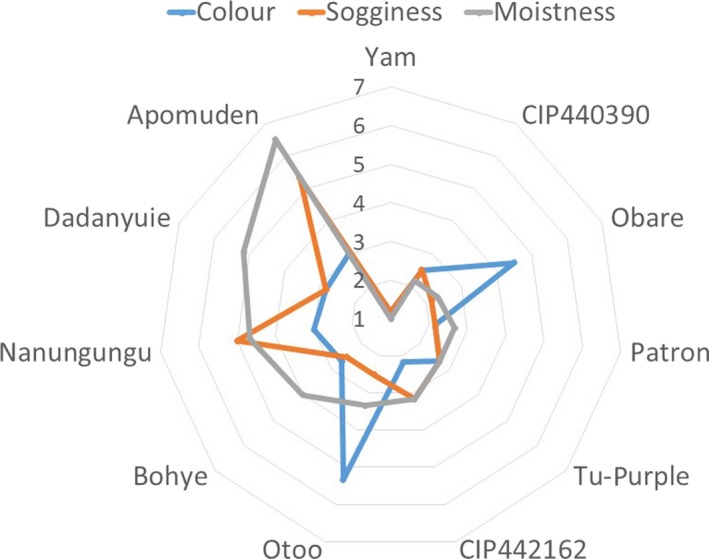
Appearance score for sweetpotato fries

The appearance score is made of color, sogginess, and moistness and is perceived mostly by sight. The moistness and sogginess were perceived by feeling in fingertips and sight as well. The color had to do with the detection of browning, sogginess the detection of oil/moisture, and moistness the detection of moisture. *Apomuden* had the highest score for moistness with an average score between 6 and 7, followed by *Dadanyuie* and *Nanungungu*. These had high moisture contents as shown in Figure 2. The lowest moistness score was seen in yam, which was the control. Moistness was found to be positively and significantly correlated with the moisture content of the fries (*r* = +0.829, *p* = 0.007).

Regarding sogginess, *Apomuden* and *Nanungungu* were regarded as soggy, which was in line with the “oily mouthcoat” in Figure 1. These two genotypes have high moisture content and hence low dry matter, and in addition to the amount of oil absorbed, the fries produced from them were soggy. Browning was seen in Otoo and *Obare* at a level of 4 and 5, which represented “somewhat brown.” The other genotypes, including the orange‐fleshed and purple genotypes, had less browning in them. Again, this indicates that the frying conditions used did not cause a lot of browning but were just enough to cook all starches in the sweetpotato. According to Teruel, Gordon, Linares, Ahromrit, and Niranjan (2015), some level of browning occurs during deep frying in oil.

#### 3.3.3 Texture score of sweetpotato fries

**Figure 3:**
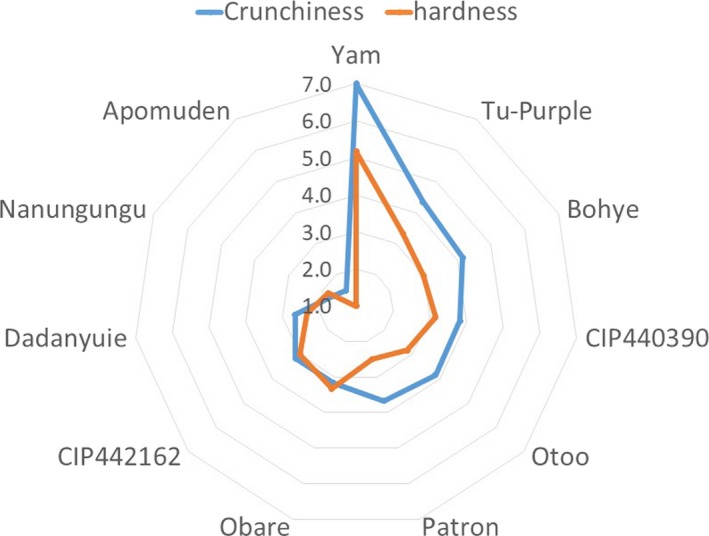
Texture score for sweetpotato fries

Texture is a very essential quality attribute for foods since it has a dominant impact on acceptability and the quality of the products (Kayacier & Singh, 2003). Regarding fries, texture is key, because the crunchiness/crispiness and hardness of a fried sample will affect its acceptability by consumers. The texture score for the sweetpotato fries was comprised of crunchiness and hardness. Yam was the crunchiest with an average score of 7, while *Tu‐Purple* and *Bohye* followed with an average score between 4 and 5, which represented “less crunchy” and “moderately crunchy,” on a scale of 1–9 (Figure 3). The least crunchy samples were *Apomuden* and *Nanungungu* with an average value less than 2, representing “soft” on the 1–9 scale developed (Figure 3).

The orange‐fleshed genotypes (*Apomuden* and *Nanungungu*) have high moisture content with low dry matter causing them to be flat/soft/stale instead of crunchy after frying, compared to yam which has high dry matter. Oner and Wall (2012) noted that the perceived softness of sweetpotato fries, due to weak crust and moist interior, was related to low dry matter and low oil contents. During frying, water, which helps maintain the texture of samples before frying, evaporates to be replaced by the oil being used to fry. However, in samples with very high moisture content, there was still some level of water present, and therefore, much oil was not absorbed. The water evaporated weakens the structure of the samples, thereby causing it to lose its texture. As a result, the sample is not able to be crunchy. This may have been the reason why low‐dry‐matter genotypes such as the orange‐fleshed genotypes used in this study are unable to be crunchy.

Hardness followed a similar trend as that of crunchiness. However, lower values were obtained (Figure 3). How hard fries may appear will affect consumer acceptability. If fries are too hard, consumers might reject it, while samples that are too soft may not be appealing to consumers. Therefore, a moderately hard sample, “a sample that is just right,” is what processors should thrive hard to achieve. This is difficult to get as it may vary with regard to the region, country, town, and even type of consumers. Therefore, a study could be conducted to determine exactly the level of hardness consumers in a particular region may prefer, in order to assist processors of fries. Sensory evaluation and texture analysis could be very important tools to use. In a study by Teruel et al. (2015), the deep‐fried fries were found to be less/moderately hard.

### 3.4 Color of fries

Color is said to be the major acceptability index of most foods by consumers due to its prominence (Pedreschi, Moyano, Kaack, & Granby, 2005). L* values beyond 50 indicate relatively lighter or brighter color, while values below 50 represent darker color (Falade & Olugbuyi, 2010). The L* values for the sweetpotato fries ranged from 43.96 to 76.02. *Bohye* (pale orange‐fleshed) had the highest L* value (76.02) followed by *Obare* (white‐fleshed) (71.51), while *Tu‐Purple* had the lowest value (43.96), which falls within the darker region (Table 3). This may be as a result of the deep purple color of the Tu‐Purple genotype. *Bohye* was observed to be brighter than the white genotypes (*Obare* and *Dadanyuie)*. This might be due to the color of the raw storage roots, and color change caused by browning during the frying process (Utomo & Rahman, 2015).

All genotypes ended up with bright colored products (L* > 50) after frying except *Tu‐Purple* which had L* value below 50. There was a significant difference (*p* < 0.05) observed among all 10 genotypes (Table 3). Meanwhile, there was no significant difference (*p* > 0.05) between *Nanungungu* and *Otoo*. *Bohye* (pale orange) had an L* value higher than that which was observed by Odenigbo et al. (2012) in Ginseng Red, a genotype with pale orange flesh color. The lightness of the white‐fleshed sweetpotato (White Travis) genotype reported by Odenigbo et al. (2012) had a value lower (64.76) than that of those in the present study, that is, *Obare* (71.51) and *Dadanyuie* (68.84) (Table 3). The variations may be due to browning of the product which is attributed to the frying temperature and time. That is, the products in this study did not brown as much as that of Odenigbo et al. (2012).

The a* and b* values express intensity of redness and yellowness, respectively (Utomo & Rahman, 2015), of the product. The a* values ranged from 0.03 to 15.55 (Table 3). These values were found to be in the range (5.84–21.84) reported by Utomo and Rahman (2015).

*Apomuden* (15.55) had the highest a* value, while *Bohye* had the lowest value (0.02). Orange‐fleshed genotypes *Nanungungu* (14.24) and *Apomuden* (15.55) had high values of a* and this could be due to their flesh colors, contributed to by the beta‐carotene content. Though *Bohye*, being pale orange, was expected to have some level of redness, it rather recorded the least value in all the genotypes. This could imply that the *Bohye* genotype tends to lose most of its beta‐carotene (which provides it with its characteristic color) during processing and maybe even frying.

All genotypes had positive values for a*, implying they can be found in the red region. The redness parameter, a*, was also highest in the orange‐fleshed sweetpotato genotype studied by Odenigbo et al. (2012). According to Odenigbo et al. (2012), samples with high a* values could indicate some level of browning, that is, in cases of long frying time with high temperatures. However, in this study, white, cream, yellow, and pale orange genotypes all had very low a* values, implying they did not brown that much, based on the frying conditions. Therefore, the orange‐fleshed genotypes having relatively higher a* values are definitely due to their flesh color, which is orange. There were significant differences (*p* < 0.05) observed among all 10 genotypes (Table 3).

The yellowness/blueness parameter, b*, was highest in *Nanungungu* (37.01) followed by *Apomuden* (33.65), which may be due to their orange flesh nature. All genotypes had positive values except Tu‐Purple which had a negative b* value (−2.86), indicating that the product is in the blue region (Table 3). This is due to its purple color. Most of the genotypes had positive b* values (thus, in the yellow region on the b* scale), which is a desirable trait in fried foods (Krokida, Oreopoulou, Maroulis, & Marinos‐Kouris, 2001). Each genotype was again found to be significantly different (*p* < 0.05) from the other.

Chroma (C*), which represents the level of saturation, ranged from 14.85 to 39.66 (Table 3). *Nanungungu* had the highest value and again was followed by *Apomuden,* while *Tu‐Purple* had the least level of saturation. The level of saturation (C*) takes into account the a* and b*; hence, this trend was observed. A significant difference (*p* < 0.05) was observed among all samples except for *Otoo* and *Patron* (Table 3).

Regarding hue angle, *h*, an angle of 90° represented a yellow hue (where b* is yellowness measured). Hue angle, *h*, is expressed in degrees: 0° (red), 90° (yellow), 180° (green), and 270° (blue). The hue angle is another parameter frequently used to characterize color in food products and has been used extensively in the evaluation of color parameter in green vegetables, fruits, and meat (Barreiro, Milano, & Sandoval, 1997). Hue angle ranged from 65.20 to 348.87 for the fried samples. Tu‐Purple recorded the highest hue angle value, while *Apomuden* recorded the lowest. Objects with higher hue angles are greener, while lower angles are more orange‐red. Tu‐Purple falls within 300° and 360°, which represents purple color indicating the actual flesh color of this particular genotype. Variations occurring in the samples, in terms of hue angle, were mainly due to their flesh colors. Hue and chroma are the qualities or attributes of any color.

The browning index ranged from 2.50 to 24.04, with *Apomuden* having the highest value while *Bohye* had the lowest value (Table 3). *Apomuden*,* Nanungungu*, and Tu‐Purple had the highest values for browning index. This may not be mainly due to the frying conditions used in this study: type of oil used, frying temperature, and time, but due to the color of the sweetpotato genotypes. Just as was seen in the L*, a*, and b* values for the orange‐ and purple‐fleshed genotypes, the browning index followed the same trend. Although all samples had browned to an extent, due to the fact that they were fried samples, the browning was not considered unappealing. A positive, strong, and significant correlation was observed between the browning index and color (*r* = +0.864, *p* = 0.01) of fried samples, observed during the sensory evaluation. The same was observed between browning index and caramel (*r* = +0.929, *p* = 0.00), also a sensory attribute during the sensory evaluation. The color followed a scale of 1 = no detection of browning to 9 = dark brown (burnt), while caramel was 1 = no caramel sensation to 9 = burnt. Since less caramel sensation was observed (values <3) and color was less than 4, it could be said that the browning index (BI) of the fried samples was appealing.

Statistically, there was no significant difference (*p* > 0.05) between *Nanungungu* and Tu‐Purple. However, there were significant differences (*p* < 0.05) among the other genotypes (Table 3). Browning of the fried samples may sometimes result from the type of oil being used. This may sometimes depend on the smoke point of the oil which results from the amount of free fatty acids in the oil (Pongsing & Chaoruangrit, 2016).

### 3.5 Beta‐carotene content of some selected fries

The β‐carotene content was determined on the orange‐fleshed sweetpotato genotypes, *Bohye, Apomuden*, and *Nanungungu*, for both raw roots and fries. The results for the beta‐carotene content are shown in Table 4. *Bohye* had the lowest beta‐carotene content (330.76 μg/100 g), while *Apomuden* had the highest (6,205.07 μg/100 g). After frying, the beta‐carotene content of all the sweetpotato genotypes decreased. That of *Bohye* almost decreased by half, while that of *Apomuden* decreased by about 31% (Table 4). *Nanungungu* was the only orange‐fleshed genotype that was able to retain most of its beta‐carotene after frying. Its beta‐carotene content only decreased by about 13% (Table 4). Therefore, even though *Apomuden* has the highest beta‐carotene content among all orange‐fleshed genotypes of sweetpotato in Ghana, it lost a significant fraction of it after frying. Fried *Nanungungu* therefore has the highest beta‐carotene content among all the fries from the orange‐fleshed genotypes.

**Table 4:** Beta‐carotene content of selected raw and fried sweetpotato

Genotype	Beta‐carotene content (μg/100 g)		% loss of beta‐carotene
	Raw sample	Fried sample	
*Bohye*	330.76 (12.84)	184.32 (2.19)	44.27
*Apomuden*	6,205.07 (39.84)	4,281.11 (30.05)	31.01
*Nanungungu*	5,715.45 (33.18)	4,961.23 (51.44)	13.20

*Notes*

Values are represented as mean (standard deviation).

Ukpabi, Ekeledo, and Ezigbo (2012) also reported a similar effect when some selected yellow‐ and orange‐fleshed sweetpotato genotypes were fried. A loss ranging from approximately 5% to 84% was reported. It is therefore a natural occurrence when beta‐carotene is lost after frying. Beta‐carotene is known to be nonpolar, dissolving in nonpolar solvents, such as the oil used in frying. Moreover, they are also heat‐labile, and as a result, could have been decomposed by the heat.

According to the Institute of Medicine (2001), based on calculations by Vidailhet et al. (2017), consuming 3.6 to 18 mg of beta‐carotene daily will maintain blood levels of beta‐carotene in the range associated with lower risk of chronic disease. The level of beta‐carotene content remaining for *Apomuden* and *Nanungungu* after frying was found within this range, which therefore indicates that the consumption of the sweetpotato fries made from *Apomuden* and *Nanungungu* could maintain the levels of beta‐carotene in the blood.

## 4 CONCLUSION

The frying conditions chosen were suitable for sweetpotato fries. The starch content of the genotypes used ranged from 10.12% in *Apomuden* to 19.79% in *Bohye*. However, dry matter ranged from 24.05% in *Apomuden* to 38.25% in *Dadanyuie*. The orange‐fleshed genotypes *Apomuden* and *Nanungungu* after frying were considered to be sweeter than the other genotypes. Caramel and starch/rawness sensation detected for all genotypes were very low, indicating the frying conditions were suitable for frying sweetpotatoes. On average, starch/rawness values were less than 2 on a 1–9 intensity scale. Oily mouthcoat, moistness, and sogginess were detected in mostly the orange‐fleshed genotypes of sweetpotato: *Apomuden* and *Nanungungu*. This may have been influenced by the level of moisture in these genotypes. Regarding crunchiness, Tu‐Purple, *Bohye*, and CIP440390 were considered to be moderately crunchy, compared with other genotypes. Fat content was highest in CIP442162 and least in *Dadanyuie*, while moisture content was highest in *Apomuden* and least in CIP440390. Compared to yam, the fat content absorbed by the sweetpotato genotypes was moderate. Browning index was highest in the orange‐fleshed and purple genotypes, but this was mostly due to their flesh colors and not the frying conditions. Among the orange‐fleshed genotypes, *Nanungungu* retained more beta‐carotene after frying. *Tu‐Purple, Bohye*, and *CIP440390* produced moderately crunchy fries and had the highest score for desirable attributes compared with the other genotypes. Therefore, *Tu‐Purple, Bohye*, and *CIP440390* could be promoted in commercial production of fries for enhanced utilization of developed sweetpotato genotypes.

## CONFLICT OF INTEREST

The authors declare that they do not have any conflict of interest.

## AUTHOR CONTRIBUTIONS

Damian Laryea was part of a team that designed the project, analyzed the data, and drafted the manuscript. Debora Koomson took part in experiments conducted, interpretation of results, and drafting of the manuscript. Ibok Oduro was part of the team that designed the study, supervised the study, and interpreted the results. Edward Carey supervised the study and helped with interpretation of data obtained.

## ETHICAL REVIEW

This study does not involve any human or animal testing.
